# Management of Comminuted Fracture of Mandible Using Titanium Mesh

**DOI:** 10.7759/cureus.35799

**Published:** 2023-03-05

**Authors:** Tejaswini P Sri Surya, Sravya Jaggannagari, Reshma Priyanka Danam, Shreya Colvenkar, Aditya Mohan Alwala

**Affiliations:** 1 Department of Oral and Maxillofacial Surgery, Malla Reddy Institute of Dental Sciences, Hyderabad, IND; 2 Department of Oral and Maxillofacial Surgery, Panineeya Mahavidyalaya Institute of Dental Sciences, Hyderabad, IND; 3 Department of Oral Medicine and Radiology, Panineeya Mahavidyalaya Institute of Dental Sciences, Hyderabad, IND; 4 Department of Prosthodontics, MNR Dental College and Hospital, Sangareddy, IND; 5 Department of Oral and Maxillofacial Surgery, MNR Dental College and Hospital, Sangareddy, IND

**Keywords:** mandible, titanium, mandibular fractures, titanium mesh, comminuted fractures

## Abstract

Comminuted mandibular fractures are common following a high-velocity injury to the face and jaws. The inherent nature of injury and damage to the underlying hard and soft tissues often complicate the management of comminuted fractures. Traditionally, comminuted fractures were managed by closed reduction and external skeletal fixation. Titanium mesh serves as an excellent alternative in the management of comminuted mandibular fractures. The present case report presents the successful management of comminuted mandibular fractures using titanium mesh.

## Introduction

Mandibular fractures occur either independently or along with other facial fractures following maxillofacial trauma. Comminuted mandible fractures are relatively rare and occur as a sequela following a high velocity or high projectile impact from sports injuries, occupational injuries, assaults or missile injuries, and gunshot wounds [1]. Unlike in other fractures, the fracture segments are multiple and arranged in a haphazard manner. Lack of adequate skin and soft tissue secondary to injury further complicates its management.

Direct fixation in such cases becomes challenging mainly due to the fear of devitalizing the bone and soft tissue while gaining surgical access and the lack of sufficient bone for screw fixation to act as a stable base. Treatment protocols mainly focus on aligning the fracture segments as conservatively as possible to preserve the blood supply. Ellis and his colleagues treated 198 patients with comminuted mandibular fractures and proposed that in gross comminution cases like gunshot wounds and missile injuries with severe soft tissue disruption, the primary treatment objective is to maintain the spatial relationship of the fractured fragments until healing occurs [2]. Scolozzi and Richter advocated that the pre-requisite for sound bone healing and low infection rate in comminuted fractures is the absolute stability of the fracture after reduction and the ability of fixation to support full functional loads of the mandible without deformation [3].

Titanium mesh and titanium trays serve as excellent adjuncts as they are biocompatible, allow sufficient space for bone graft placement, and require minimum screws to hold the tray/mesh in a position [4,5]. When used judiciously they shortened the recovery period and greatly improve the functional outcomes after surgery. Custom-made titanium mesh and trays are available that are adjusted intra-operatively for accurate adaptation over the surgical defect. This case report presents the successful management of comminuted mandibular fracture using titanium mesh.

## Case presentation

**Figure 1:**
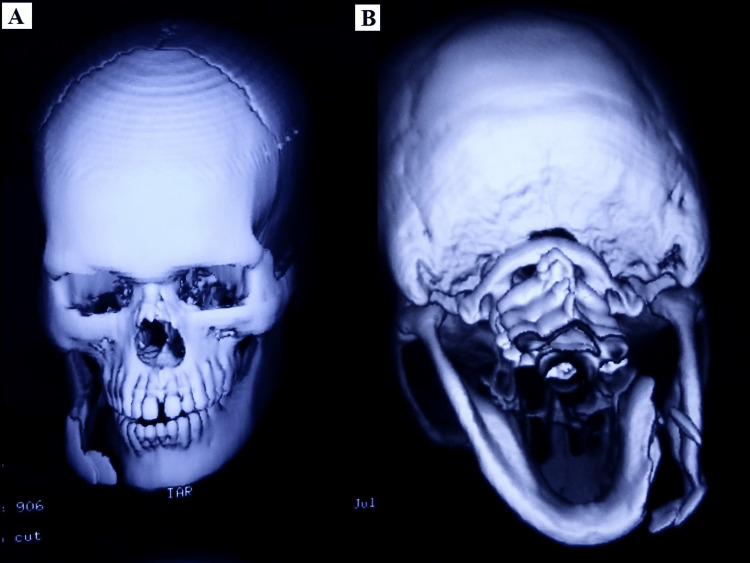
Pre-operative CT scan images showing comminuted body fracture of mandible. (A) Frontal view. (B) Posterior-anterior view.

A 28-year-old male patient reported with a broken lower jaw to the Department of Oral and Maxillofacial surgery. History revealed the patient had an accidental borewell blast and sustained a severe impact to the right lower jaw with gross comminution of hard and soft tissues. CT scan reports were conclusive of a comminuted fracture of the right body of the mandible (Figures 1A, 1B).

**Figure 2:**
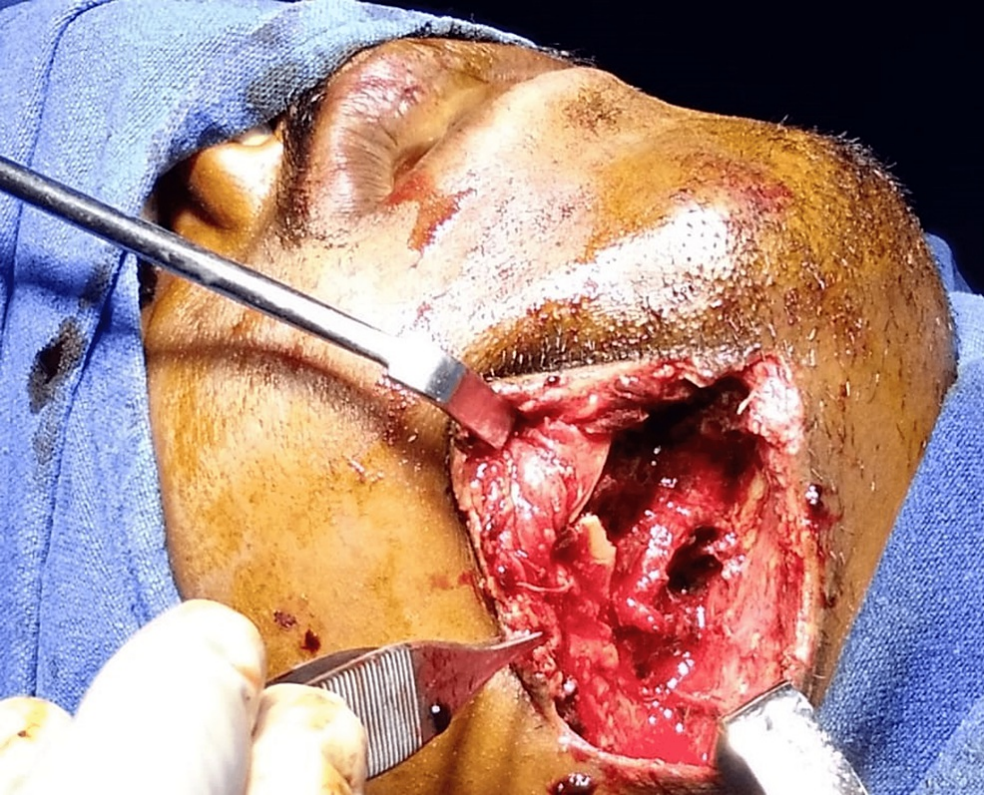
Intra-operative image showing surgical access to the fracture site

Open reduction and internal fixation of the comminuted fracture with Titanium mesh under general anesthesia was planned. An extra-oral approach was preferred to gain access to the fracture site through the existing skin laceration (Figure 2).

**Figure 3:**
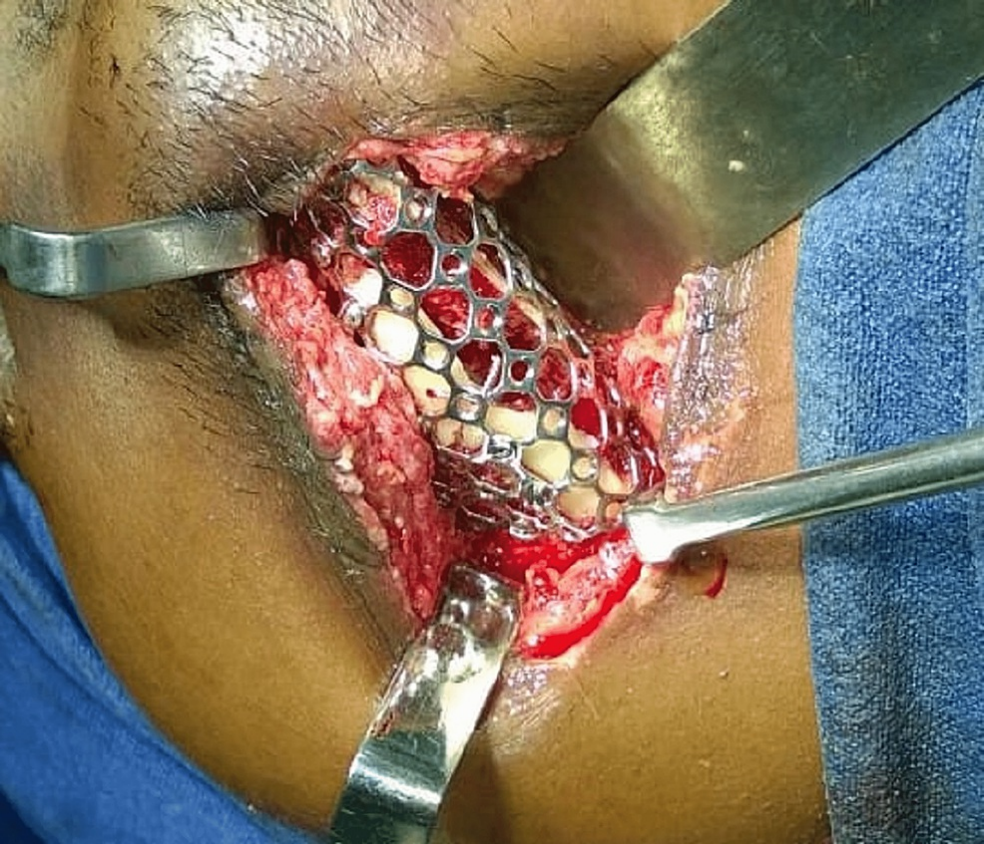
Intra-operative image showing titanium mesh in position

After adequate exposure and wound debridement, multiple fracture fragments were identified in the right body of the mandible at the fractured site. A custom-made titanium mesh was contoured and adjusted till posterior restore the continuity defect (Figure 3). The titanium mesh was extending in the molar region as the comminution was there till posterior.

**Figure 4:**
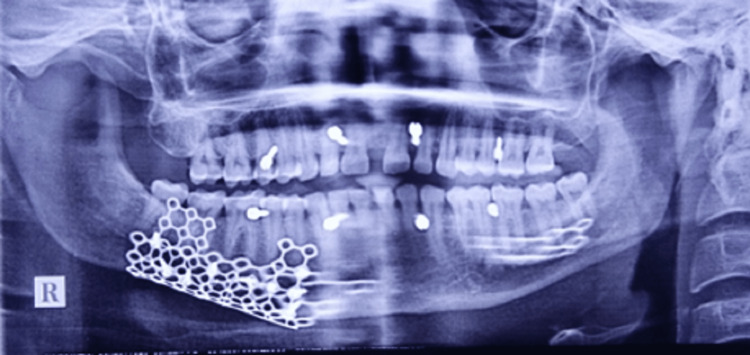
Post-operative radiograph showing Titanium mesh

Following minimal periosteal stripping, the comminuted fragments were realigned and secured in position with the previously contoured titanium mesh and screws (Figure 4). Intermaxillary fixation was done only intraoperatively. Postoperatively occlusion was intact; hence, intermaxillary fixation wires cannot be seen in OPG.

Following open reduction and fixation, the fracture site was immobilized with a maxillo-mandibular fixation with screws for a period of two weeks. Post-operative healing was uneventful, and the patient was discharged the next day.

## Discussion

Comminuted fractures usually present as compound or complex fractures with direct communication to the external environment. Traditionally, comminuted fractures were treated like a bag of bones, and treatment objectives mainly focused on preserving the remaining vitality of hard and soft tissue structures.

The success of open reduction and internal fixation with plates and screws makes it the most popular treatment for fractures. Stainless steel and titanium plating systems are routinely used for reconstruction in maxillo-facial trauma. Titanium is biologically compatible, relatively inert, flexible, and has an elastic modulus like that of bone. Numerous research studies compared the efficacy of titanium and stainless bone plates. Taheri et al. concluded that when compared to stainless steel titanium screws generated lower stresses at the fracture site during the physiological loading of bone [6]. Uhthoff et al. in their study concluded that titanium-plated bones showed less cortical bone loss and thickness with greater regain in bone density after plate removal compared to stainless steel bone-plated bones [7]. Seligson et al. observed that titanium-plated bones had higher strength compared to stainless steel-plated bones after plate removal [8]. The study by Lujan and his colleagues suggested that titanium-plated bones allowed better callus formation as compared to stainless steel-plated bones [9].

Titanium mesh and titanium trays are preferable over titanium mini plates when indicated [4-6]. When compared to titanium mini plates, titanium mesh offers superior 3-dimensional stability, and greater malleability hence has better-handling properties to be contoured, offers a semi-rigid fixation with less stress shielding effect, and promotes faster healing by allowing micro-movements at the fracture segments. In ridge augmentation procedures, porous titanium mesh has shown excellent results in bone regeneration when used alone or as an adjunct with resorbable membranes and bone morphogenic proteins. The presence of porosity or channels in the mesh ensures uncompromised blood supply to the underlying bone and soft tissues, thus, maintaining the vitality of these structures.

Bioresorable plates have been designed to overcome the shortcomings of metal implant systems. But they are not routinely preferred due to their inherent lack of sufficient mechanical strength to allow stable fixation and the risk of adverse foreign body inflammatory response during biodegradation of the implant in the body during healing.

One of the main complications of titanium mesh is mesh exposure following soft tissue dehiscence leading to infection and fistula formation. Nazzal et al. observed that with the increase in implantation time of metal mini plates, there is a significant increase in inflammatory cells and thus advocated the removal of non-functional mini plates after bone healing [10]. Debate still exists regarding the removal of these metal mesh, plates, and screws after the completion of fracture healing. The patient was evaluated regularly for any signs of hardware infection, failure, toxicity, or foreign body reaction. The patient showed good signs of healing on regular follow-ups. Only if the patient had complications second surgery for plate and mesh removal would be indicated in the future.

## Conclusions

Mandibular comminuted fractures present a significant challenge to the reconstructive surgeon. While the debate for an ideal reconstruction material with clinically desirable properties continues, titanium metal plates, mesh, and implant systems gained enormous popularity amongst maxillofacial surgeons worldwide due to their excellent biocompatibility, mechanical rigidity, and low corrosion degradation as compared to stainless steel. titanium mesh is an excellent adjunct for the management of complex comminuted fractures when indicated. This case report presents the successful management of comminuted mandibular fracture using titanium mesh.
